# The effects of MYC on tumor immunity and immunotherapy

**DOI:** 10.1038/s41420-023-01403-3

**Published:** 2023-03-25

**Authors:** Jiajin Li, Tingyu Dong, Zhen Wu, Dacheng Zhu, Hao Gu

**Affiliations:** 1 Department of Pediatrics, Second Clinical School of Medicine, Anhui Medical University, Hefei, China; 2 Department of Clinical Medicine, First Clinical School of Medicine, Anhui Medical University, Hefei, China; 3 Department of Immunology, School of Basic Medical Sciences, Anhui Medical University, Hefei, China

**Keywords:** Oncogenes, Tumour immunology

## Abstract

The oncogene MYC is dysregulated in a host of human cancers, and as an important point of convergence in multitudinous oncogenic signaling pathways, it plays a crucial role in tumor immune regulation in the tumor immune microenvironment (TIME). Specifically, MYC promotes the expression of immunosuppressive factors and inhibits the expression of immune activation regulators. Undoubtedly, a therapeutic strategy that targets MYC can initiate a new era of cancer treatment. In this review, we summarize the essential role of the MYC signaling pathway in tumor immunity and the development status of MYC-related therapies, including therapeutic strategies targeting MYC and combined MYC-based immunotherapy. These studies have reported extraordinary insights into the translational application of MYC in cancer treatment and are conducive to the emergence of more effective immunotherapies for cancer.

## Facts

- MYC regulates a variety of biological processes, including cell proliferation, survival, aging, and differentiation.
- The abnormal expression of MYC is one of the most common carcinogenic events in human cancer.
- MYC modulates immune effector cells and immune regulatory factors in the tumor immune microenvironment.
- Targeting MYC provides a huge research prospect for tumor immunotherapy.

## Open questions

- How MYC inhibits immune cells and promotes tumor escape in the tumor microenvironment?
- What are the key molecules in the MYC pathway mediating antitumor immunity?
- How to target MYC for retarding tumor growth?

## Introduction

Studies have found that the fundamental cause of tumor occurrence and development is genetic variation, which involves two types of genes in cells, namely, proto-oncogenes and tumor suppressor genes. MYC was the first proto-oncogene that was found to be amplified in tumor cells, and its overexpression is involved in tumor-related processes. As early as 1981, scientists proposed for the first time that the integration of virus promoter can drive the expression of cell MYC (c-Myc) and lead to cancer [1], and MYC was first found in human Burkitt lymphoma in 1982 [2]. Since then, MYC has become a hot gene in the field of tumor research, and even now, research on this oncogene is still ongoing. Based on studies of MYC transcriptional repressors, subsequent studies on a series of effects of MYC in tumor models and analyses of the transcriptional targets of MYC oncoproteins have been performed [3, 4]. These studies, as well as current popular studies of MYC immunotherapy, have led scientists to gradually reveal the mystery of MYC.

The Myc family includes three members: MYC, MYCN, and MYCL [5, 6]. As a member of the Myc family, MYC is an important proto-oncogene that is overexpressed in a variety of human tumors where it acts as a transcriptional regulator. The MYC gene is localized to chromosome 8, and its carboxyl terminus contains the b-HLH-Zip domain, which combines with a similar domain, MYC-associated factor X (MAX); these domains can then combine with the E box located on a gene promoter and initiate transcription of the corresponding gene. Moreover, downstream effectors of MYC coordinate a wide range of biological functions, including cell proliferation, differentiation, and immune surveillance [7]. Therefore, it is not surprising that the MYC signaling pathway is considered a potential therapeutic target for cancer therapy due to its role in tumors. Furthermore, increasing evidence suggests that MYC hyperactivation can mediate tumor-induced immunosuppression at multiple levels [8, 9]. Surprisingly, tumor regression associated with tumor microenvironment remodeling occurs when MYC expression is silenced in multiple tumor models [10, 11]. Despite numerous challenges in MYC-related immunotherapy, continuous major breakthroughs in recent years have given scientists unwavering confidence in targeting MYC to treat cancer [12, 13]. Undoubtedly, an in-depth understanding of the mechanism of action of the MYC gene family in tumor immunity and its application in tumor immunotherapy will be crucial to the development of effective cancer therapies.

This review first briefly introduces the structure and function of MYC and its function in driving tumor immunosuppression to link MYC targets with tumor immunotherapy and introduces multiple cancer immunotherapies from different perspectives.

## MYC-driven tumor immune suppression

**Fig. 1:**
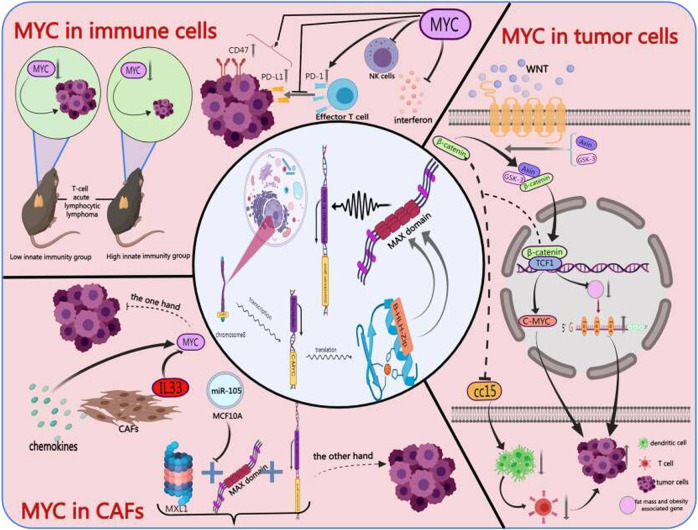
MYC in cells. Activation of the WNT/β-catenin and MYC pathways can induce tumor cell proliferation and MYC can participate in immune cell-related activities to inhibit innate and adaptive immune responses. In addition, MYC can be activated by several cytokines secreted by CAFs, including IL-33 and chemokine, which leads to the suppression of tumor immunity.

The composition of the tumor microenvironment (TME) varies among tumor types, but hallmarks mainly include the presence of tumor cells, tumor-infiltrating immune cells (such as macrophages and dendritic cells), smooth muscle cells, endothelial cells, and cancer-associated stromal cells (such as cancer-associated fibroblasts (CAFs) [14]. Over time, increasing numbers of researchers have realized that MYC can drive tumor immunosuppression by interacting with other molecules in the TME [15] (Fig. 1). The overactivation of MYC in the TME might have a remarkable influence on cancer development through different mechanisms (some details are discussed below).

### In tumor cells

Numerous studies have shown that the MYC-dependent pathway in tumor cells can indirectly affect the expression of cytokines in the TIME, such as the reduction in the expression of interleukin-2 (IL-2), interferon-γ (IFN-γ), and perforin [16] and the increase in the expression of interleukin-6 (IL-6) [17]. For example, nuclear Aurora kinase A (AURKA), a serine/threonine kinase, participates in mitosis and can increase the expression of programmed death-ligand 1 (PD-L1) via an MYC-dependent pathway and then inhibit IFN-γ expression. In this way, immune evasion by tumors can occur [16].

In tumor cells, other signaling pathways often interact with MYC to provide strong support for tumor progression, especially the WNT/β-catenin pathway [18]. Abnormal WNT/β-catenin pathway can be observed in a variety of cancers like breast cancer, colorectal cancer, and hepatocellular carcinoma [19–21], which often acts as the upstream carcinogenic signal of MYC and participates in the process of proliferation, apoptosis, and angiogenesis [22]. Notably, the interaction between the WNT/β-catenin pathway and MYC may be important for tumor immune suppression: (1) Both the WNT/β-catenin pathway and MYC are frequently activated in the same tumor cells and share common targets that participate in cell proliferation, anti-apoptosis, and metastasis. (2) The WNT/β-catenin pathway can mediate the downregulation of fat mass and obesity-associated gene, which significantly enhances the m6A level in MYC mRNA and ultimately induces tumor cell proliferation and tumorigenesis [23]. (3) The MYC/ β-catenin cooperation depends on β-catenin to promote the immune escape of tumor cells: especially β- catenin inhibits the expression of chemokine Cc15, which leads to recruitment defect of dendritic cells and impaired T cell activity[24]. (4) The classic oncogene MYC induces immune escape by inhibiting innate immunity, and when the WNT/β-catenin pathway and the downstream molecule MYC are inhibited, especially MYC, tumor proliferation and metastasis may also be inhibited [25]. These studies demonstrate the importance of the WNT/β-catenin pathway and MYC in tumor immunity.

### In immune cells

In the TME, MYC participates in immune cell-related activities to inhibit both innate and adaptive immune responses [26, 27]. Here, we briefly discuss multiple immune-related functions of MYC in the tumor immune environment and some recent research progress.

Briefly, overactivation of MYC in innate immune cell subsets may be detrimental to the production of proinflammatory mediators, such as interferon, may increase immunosuppressive cytokine production, and may restrain the tumor-killing activities of effector cells, such as NK cells [27, 28]. For instance, in the MYC transgenic mouse model with T cell acute lymphocytic lymphoma, the inactivation of MYC induced continuous tumor regression in the wild type, but it did not occur in the immunocompromised hosts [29], which indicates that MYC may regulate the tumor microenvironment by regulating the innate immune system and regulating cytokines. Overactivation of MYC in adaptive immune cell subsets mainly reduces the killing effect on the tumor by inhibiting effector T cells, in which MYC regulates the expression of CD47 and PD-L1 in various types of tumor cells [8, 30]. Tumor-associated macrophages (TAMs) are abundant within the TIME where they participate in immunosuppression [31]. Programmed cell death protein 1 (PD-1) is an immune checkpoint receptor that is upregulated on activated T cells to induce immune tolerance. Blocking PD-1-PD-L1 in vivo can increase macrophage phagocytosis and effectively reduce tumor growth [31, 32]. MYC affects related immune activities by regulating innate and adaptive immunity in the TIME to promote tumor proliferation.

### In CAFs

Most CAF subpopulations usually exert cancer-promoting effects and participate in multiple stages of tumor development in a variety of ways [33, 34]. On the one hand, some studies have shown that MYC can be activated by some cytokines in CAFs, including IL-33 and chemokines, to inhibit tumor immunity [35]. On the other hand, miRNA headed by miR-105, because of its high cancer-to-normal secretion ratio in MCF10A extracellular vesicles, can reduce MXI1, a protein that can form a heterodimer with MAX. This MYC-MAX compound inhibits transcription of the MYC promoter, increases MYC protein levels in CAFs, and finally induces an MYC-dependent metabolic program to participate in tumor growth and development [36, 37]. Moreover, a recent study revealed that CAFs may signal in a paracrine manner to enhance the expression of MYC in tumors to drive tumorigenesis [38].

## MYC mediates crosstalk between tumor cells and different immune cell subsets in the TME

**Fig. 2:**
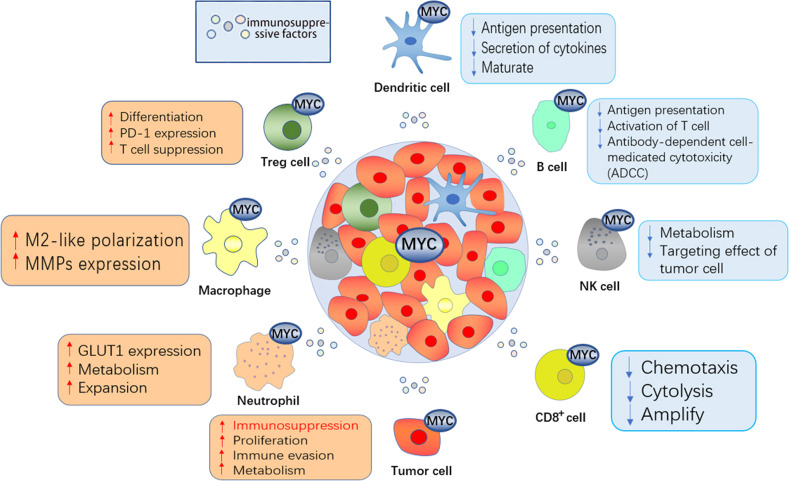
MYC induces immunosuppression in the TIME. MYC is good for the accumulation and enrichment of immunosuppressive Treg and B cells and the polarization of M2-like macrophages, which triggers immune evasion. On the contrary, MYC can achieve an immunosuppressive effect by affecting CD8 + T cells, NK cells, and neutrophils and causing suppressive antitumor cytolysis. In addition, dysregulated MYC expression in tumor cells supports multiple hallmarks of cancer, such as the secretion of immunosuppressive factors, which can downregulate the function of other immune cells and help tumor cells escape immune attack. Overall, MYC induces immunosuppression in the TIME, thereby promoting tumor progression and reducing antitumor immunity.

Hyperactivation of MYC initiates tumor-induced immune suppression through crosstalk between tumor cells and different immune cell subsets in the TME, such as T cells, B cells, macrophages, natural killer cells, dendritic cells (DCs), and neutrophils (Fig. 2). Here, we briefly mention the relevant content.

### MYC mediates crosstalk between tumor cells and T cells

CD8 T cells are critical for the establishment of antitumor immunity, and canonical BRG1/BRM-associated factor (cBAF) and MYC frequently co-associate with two daughter cells asymmetrically during the first division of activated CD8 T cells. Daughter cells with high MYC and high cBAF show a cell fate trajectory towards T(eff) cells, whereas daughter cells with low MYC and low cBAF differentiate preferentially towards T(mem) cells [39]. It can be applied to manipulate cBAF complexes and MYC in the early stages of T cell differentiation, thereby improving cancer immunotherapy. Forkhead box O (FOXO) proteins are a common family of transcription factors in mammals [40]. The FOXO1 transcription factor inhibits MYC signaling [41], and FOXO3a, a transcription factor inhibited by the phosphatidylinositol 3-kinase/Akt pathway and upregulated in hypoxia, has become an important negative regulator of MYC function [42]. FOXO plays an important role in regulating specialized lymphocyte function and maintaining T cell quiescence. Enhanced Foxo1 activity disrupts CD4 T and Treg cell homeostasis [43]. Therefore, it can play a role in regulating the activity of MYC and indirectly through the regulation of FOXO, thereby affecting the proliferation of CD4 T cells. In addition, the crosstalk between MYC and tumor cells and Tregs also deserves attention. Existing studies have shown that MYC is central for coordinating Treg accumulation, transitional activation, and metabolic programming [44]. For example, when MYC is overactivated, it can promote glycolysis in tumor cells, which results in a low-glucose environment. In this environment, Tregs can actively absorb lactic acid and promote the nuclear factor of activated T cell 1 (NFAT1) translocation into the nucleus, which enhances PD-1 expression and inhibits the PD-1-expressing effector T cells [45]. In this way, the tumor continues to develop.

### MYC mediates crosstalk between tumor cells and B cells

In the TIME, tumor-infiltrating B lymphocytes (TIBs) exist in all stages of cancer and participate in shaping tumor development. On the one hand, some studies have shown that TIBs can induce and maintain antitumor activity [46]. On the other hand, other researchers have found that B cells may play a tumor-promoting role, and B cells with tumor-promoting effects are defined as regulatory B cells (Bregs) [47]. Among hematopoietic malignancies, genomic abnormalities involving the MYC gene are almost always found in B-cell lymphomas but rarely in T cell lymphomas. Oncoprotein B-cell lymphoma 6 (BCL6) is a direct inhibitor of MYC during germinal center (GC) responses. BCL6 binds to the promoter region of MYC in pre-B cells and differentiated B cells [48]. In GCs, when B cells interact with antigens and T-helper (Th) cells, they are transiently expressed due to transcriptional repression of BCL6 by inhibitory mechanisms, including via BCR, IL-2, and interferon regulatory factor 4 (IRF4), which are induced upon CD40 activation [49]. In addition, MYC is involved in the histological transformation of indolent mature B-cell malignancies to aggressive diffuse large B-cell lymphoma (DLBCL) [50].

### MYC mediates crosstalk between tumor cells and macrophages

Macrophages, as part of the mononuclear phagocytic immune system, play an important role in tumor immunity. Generally, macrophages are divided into two categories: M1-like macrophages and M2-like macrophages. Macrophages found in the TIME are usually called tumor-associated macrophages (TAMs), which are mostly the M2 phenotype [51]. One study showed that TAMs are more prone to glycolytic metabolism in the hepatocellular carcinoma (HCC) tumor microenvironment, which depends on the activation of WNT2b/β-catenin/MYC signaling [52]. In this way, lactic acid derived from tumor cells can activate NRF2 in macrophages, which in turn affects macrophage M2 polarization [53]. In addition, NRF2 is a downstream regulator of MYC and may be involved in regulating the metabolism and polarization of HCC-TAMs [52]. Furthermore, TAMs promote tumor progression and invasion by secreting matrix metalloproteinases (MMPs) [54], and of these, MMP1 can activate cdc25a/CDK4-cyclin D1 and p21/cdc2-cyclin B1 complexes to accelerate the growth of colon cancer cells via the regulation of MYC expression [55].

### MYC mediates crosstalk between tumor cells and NK cells

Natural killer (NK) cells, which participate in the immune response to tumors by killing target cells and producing cytokines, have strong proinflammatory and antitumor activity [56]. One study indicated that, in a variety of cancers, activated MYC in tumor cells participates in miR-29c-3p-targeted CD276 signaling to mediate immune surveillance, thereby avoiding NK-cell cytotoxicity [57]. It is worth noting that CD276 is a recognized costimulatory immune checkpoint that exerts antitumor effects and that can inhibit tumor growth and metastasis [58, 59]. In recent years, however, many disputes have arisen about the role of CD276. For example, the deletion of CD276 in T cells can inhibit T cell proliferation and T-helper cell-mediated immune response [60]. At the same time, the knockdown of CD276 expression reduces the adhesion, migration, and invasiveness of tumor cells [61]. This study on NK cells in tumors also suggests that CD276 acts as an oncogene, which may provide a new direction and therapeutic target for NK-cell-based immunotherapy. In addition, MYC is probably essential in the early stage of NK-cell activation. When NK cells are in glutamine-restricted tumor microenvironments, the proliferation of NK cells is inhibited, which leads to a reduction in metabolism and antitumor function [62].

### MYC mediates crosstalk between tumor cells and DCs

Dendritic cells have long been the focus of cancer immunotherapy because of their role in inducing protective adaptive immunity [63]. MYC is an important transcription factor associated with DC development and differentiation, DC maturation and metabolism, and the regulated phenotype of MPLA-activated DCs [64]. More importantly, CD47 is a more important “mediator” of MYC-mediated crosstalk between tumor cells and dendritic cells [8]. In various types of cancer cells and solid tumor tissues, high CD47 protein levels, which are mainly triggered by the transcription factor MYC, have been observed. Blockade of CD47 interaction with SIRPα ligands on phagocytes enhances cancer cell clearance by phagocytes such as macrophages and dendritic cells (DCs) to activate innate immune responses, a process that promotes antigen-presenting cells antigen cross-presentation, which leads to the initiation of T cells, which activate the adaptive antitumor immune response [65, 66].

### MYC mediates crosstalk between tumor cells and neutrophils

As a line of defense against invading pathogens, neutrophils play an important role in human immunity, including in cancer [67]. Substantial evidence indicates that tumor-associated neutrophils (TANs), as a tumor-related subpopulation, are important components of tumor-enhancing inflammation in many types of cancer [68, 69], especially malignant tumor disease of the hematopoietic system. Chronic myelomonocytic leukemia (CMML), a heterogeneous stem cell malignancy, can be divided into stages CMML-0/1/2 according to the proportion of downstream blasts and promonocytes [70]. Neutrophil-primed progenitor genes and MYC transcription factor regulators influence tumor progression and are prominently expressed in stem cells from CMML-1 patients [71]. Similarly, MYC is overexpressed in acute myeloid leukemia (AML) and chronic lymphocytic leukemia (CLL) and is able to inhibit the expression of the circadian clock gene Period2 (Per2), which is highly expressed in neutrophils where it enhances antitumor proliferation [72]. Furthermore, the voltage-gated sodium channel Nav1.5 has been reported to be highly upregulated in a variety of cancers, such as breast, ovarian and cervical cancers [73–75]. The expression level of Nav1.5 is closely related to the proportion of neutrophils and lymphocytes. Researchers found that Nav1.5 knockdown could inhibit the expression of β-catenin, cyclin D1, and MYC in the WNT/β-catenin signaling pathway, thus promoting the apoptosis of tumor cells and effectively inhibiting the migration and invasiveness of tumor cells [76].

## Targeting MYC for tumor immunotherapy

**Fig. 3:**
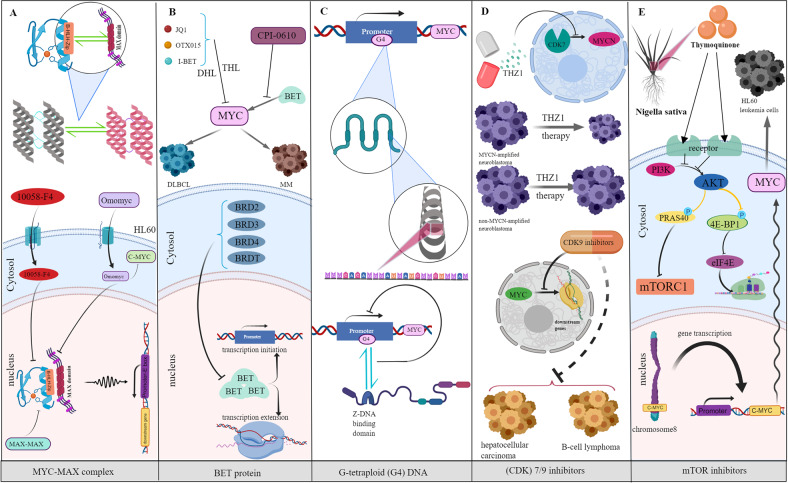
Immunotherapy targeting MYC mainly includes MYC as a direct or indirect target. The function of MYC is closely related to MYC-MAX, bromodomain and extraterminal (BET), and G-tetraploid structures. **A**–**C** MYC as a direct target includes disrupting the stability of MYC-MAX, BET inhibitors, and stabilizing G-tetraploid motifs. **D**, **E** MYC can also be indirectly inhibited by inhibiting CDK7/9 or mTOR, thus exerting immunotherapeutic effects.

Many potential strategies have been developed for compounds that directly or indirectly inhibit the MYC protein (Table 1 and Fig. 3).

**Table 1:** Potential strategies with compounds for the direct or indirect inhibition of MYC protein.

Target	Mechanism	Compound	Clinical trial
MAX	Blocking MYC–MAX interaction	OmoMYC	Phase I/II
		10074-G5	Preclinical
		KJ-Pyr-9	Preclinical
		MYCMI-6	Preclinical
	Stabilizing MAX homodimers	KI-MS2-008	Preclinical
BRD4 (and other BET proteins)	Epigenetic silencing	JQ1/TEN-010	Preclinical
		BI 894999	Phase I
		SF1126	Phase I
		CPI-0610	Phase I/II
		INCB057643	Phase I/II
G-quadruplexes	Stabilizing MYC G4 and repressing its transcription	GQC-05	Preclinical
		Cz1	Preclinical
		IZCZ-3	Preclinical
		BMH-21	Preclinical
		CX-3543	Phase II
PP2A	Driving dephosphorylation of specific pathogenic substrates such as c-Myc	DT-061/(AZD6244)	Preclinical
		OP449	Preclinical
		SMAPs	Preclinical
		FTY720	Preclinical
		LB-100	Phase I/II
Pin-1	Inhibiting Pin-1	Juglone	Preclinical
		KPT-6566	Preclinical
		Sulfopin	Preclinical
		ATRA	phase I/II/III/IV

### Direct MYC inhibitory strategies

Consistent with the role of MYC as a driver of tumorigenesis, inhibition of its expression or function can reverse tumorigenesis. MYC is a direct therapeutic target that faces two pressing challenges: (a) Most MYC is disordered in nature; (b) MYC has no obvious small molecule binding pocket [77]. The existing therapeutic strategies of directly targeting MYC in tumors mainly include blocking the interaction between MYC and MAX using BET inhibitors and stabilizing the G-quadruplex (G4) motif in the MYC promoter [77–79].

The interface of the MYC-MAX dimer is a parallel left-handed four-helix bundle, and each monomer is composed of two R helices separated by a ring [80]. The MYC-MAX complex is necessary for MYC to bind to DNA and then activate target gene transcription [5]. Targeting the stability of MYC-MAX has been widely used to inhibit MYC signaling. The compound 10058-F4 can destroy the MYC-MAX complex in HL60 cells [81]. Similarly, it was predicted that Omomyc (One of the most extensively investigated MYC inhibitors) had the potential to disrupt the MYC-MAX network and act as an inhibitor of c-Myc function [82]. MAX can also form homodimers with itself, and these complexes act as antagonists of MYC/MAX by competing for common DNA targets.

Chromatin-dependent signal transduction of RNA polymerase can specifically inhibit MYC, among which the bromodomain and extraterminal (BET) protein is related to transcription initiation and extension and is most often inhibited. BET inhibitor (BETi) proteins, including BRD2, BRD3, BRD4, and BRDT, have been shown to inhibit MYC proliferation in various tumor models [78]. JQ1, I-BET, and OTX015 can significantly reduce the proliferation of MYC in diffuse large B-cell lymphoma (DLBCL) by targeting double-hit lymphoma (DHL)/triple-hit lymphoma (THL) [83]. CPI-0610 is an effective BET inhibitor in multiple myeloma (MM) and is currently being tested in phase I clinical trial [84]. BET inhibitors combined with other drugs may have better prospects.

G-tetraploid (G4) DNA is a quadruple helix structure formed by continuous guanine-rich DNA sequences, which can block the combination of transcription factors to inhibit MYC gene transcription [79]. Some proteins inhibit MYC gene transcription by binding, promoting, or stabilizing the G4 motif. For example, the Z-DNA binding domain of adenine deaminase acting on RNA 1 (ADAR1) can directly bind to MYC G4 and stabilize its parallel strand conformation, which results in the inhibition of MYC gene expression [85]. In addition, the development of fluorescent probes with high specificity and affinity to detect MYC G4 elements will become an important experimental tool for the study of MYC tumor therapy [86, 87].

In summary, many strategies can be used to directly target MYC-driven tumors. While some BET inhibitors have entered clinical trials, most MYC-MAX inhibitors have not. The MYC G4 element has an important use in developing fluorescent probes with high specificity and affinity.

### Indirect MYC inhibitory strategies

The indirect MYC treatment strategy involves regulating the activity of signaling molecules or substances related to MYC, thereby weakening its expression in tumors. Several drugs that indirectly target MYC by triggering lethal interactions or acting on upstream signaling pathways have entered clinical trials, including cyclin-dependent kinase (CDK) 7/9 inhibitors and mTOR inhibitors [77, 88, 89].

MYC-driven cancer depends on CDK7 and CDK9. By promoting Pol II Ser5 phosphorylation by CDK7, MYC indirectly recruits RNA guanine-7 methyltransferase (RNMT) to MYC target genes, enabling the subsequent methylation of the 5′ guanine cap [90, 91]. CDK7 inhibition is a promising avenue for targeting MYC-driven transcriptional amplification. CDK9 inhibits MYC recruitment and is also a promising target in MYC-driven tumors. Cell line models of hepatocellular carcinoma and B-cell lymphoma driven by MYC overexpression showed sensitivity to CDK9 inhibitors [92, 93]. The high sensitivity of MYC to CDK7 or CDK9 inhibitors may be due to the decreased expression or stability of MYC or the inhibition of target genes regulated by MYC. In addition, since CDK7 and CDK9 influence the transcription of genes that are not regulated by MYC, targeting these kinases may lead to off-target effects. Therefore, the development of inhibitors that disrupt the interaction between the kinase and MYC protein are expected to eliminate the negative effects.

Another common way to indirectly target MYC is the use of an mTOR inhibitor. The mTORC1-dependent phosphorylation of eukaryotic translation initiation factor 4E (eIF4E) binding protein 1 (4EBP1) can block the ability of mTOR to negatively regulate the translation initiation factor eIF4E, thus promoting the translation of mRNA containing complex RNA secondary structures (such as MYC) [94]. Thymoquinone, a bioactive constituent in Nigella sativa, can potentially inhibit proliferation and induces apoptosis in HL60 leukemia cells by downregulation of c-Myc expression through inhibition of the PI3K/AKT/mTOR signaling pathways [95]. In addition, in several studies, these inhibitors exhibited significant therapeutic effects in MYC-driven cancers (including neuroblastoma, small-cell lung cancer, and multiple hematopoietic cancers) [89, 96].

Compared with direct targeted MYC therapy, indirect therapy may result in more resistance, such as off-target effects. Although many indirect inhibitors of MYC have been tested in clinical trials, problems such as drug resistance still need to be solved.

## MYC combined with immunotherapy

With the continuous development of research, immunotherapy has now become a remarkably promising method for the treatment of malignant tumors [97]. Immunotherapy based on chimeric antigen receptor (CAR)-T cell therapy has solved some problems in malignant tumor disease of the hematopoietic system, but numerous unsolved problems in solid tumors remain [98, 99]. For example, CAR-T cells are easily blocked by other immunosuppressive molecules or cells after they enter tumor tissue, and thus, they cannot play a role in solid tumors [100]. At the same time, it is worth noting that due to tumor heterogeneity and the differences among various cancers, it is difficult to find a treatment strategy that is applicable to all types of cancers [101]. Therefore, combined immunotherapy has been developed and is a promising direction to improve the outcomes of cancer treatment.

### Combined blockade of MYC and immune checkpoints

Abundant evidence suggests that MYC acts as a transcriptional amplifier; that is, it can generally increase the expression of multiple genes rather than specific target genes [102]. Dysregulation of immune signaling molecules induced by multiple signaling pathways regulated by MYC, including CTLA-4, CD47, and PD-L1, can help tumor cells directly or indirectly escape the immune response [8, 103, 104]. For instance, MYC can directly bind to the CD47 promoter to trigger its expression, after which CD47 acts as a ligand for SIRPα (signal-regulatory protein α); this triggers SIRPα phosphorylation of ITIM (immunoreceptor tyrosine-based inhibitory motif), which leads to myosin inactivation [105]. These effects promote tumor growth and phagocytosis inhibition. In addition, immune checkpoint molecules can also affect MYC expression. Shuang Qu et al. reported that PD-L1-lnc, an alternative splicing gene product of PD-L1, can promote lung adenocarcinoma (LUAD) proliferation by enhancing MYC transcriptional activity [106]. Similarly, one report showed that silencing CD47 decreased MYC expression in oral squamous cell carcinoma, which indicates that CD47 can increase MYC expression [107]. Unfortunately, it is still unclear whether the CD47-MYC-CD47 feedback loop can regulate CD47 expression.

In terms of the current research results, the combined blockade of MYC and immune checkpoints has shown surprising results. APG-115, an indirect inhibitor of MYC, can promote antitumor immune effects in cells in the TIME by downregulating some targets, such as MYC. Simultaneously, another study reported that APG-115 plus anti-PD-1 combination therapy remarkably reversed the immunosuppressive TIME into an antitumor immune environment, which led to enhanced therapeutic benefits in mice [108]. Moreover, Weiping Li et al. found that compared with using bromodomain extraterminal inhibitors (BETi) (JQ1, I-BET, and OTX015), which can downregulate CD47 expression, the therapeutic strategy with BETi and the BCL-2 antagonist ABT-199 could more effectively inhibit the expression of MYC and CD47 and significantly reduce cancer cell proliferation [83]. Undoubtedly, the combined blockade of MYC and immune checkpoints demonstrates strong therapeutic potential. However, preclinical studies that support the application of such combination therapies for cancer treatment are limited, and a large number of clinical trials are still in the testing stage.

### Combined MYC inhibitors and CAR-T

Adoptive cell therapy (ACT) is a type of immunotherapy that involves the use of immune cells to treat cancer. One of the most attractive emerging areas in ACT is the development of chimeric antigen receptors (CARs). Chimeric antigen receptors (CARs) are the core component of CAR-T, which allows T cells to recognize tumor antigens in an HLA-independent manner and enables them to recognize a wider range of target antigens than native T cell surface receptors (TCRs) [109].

The transcription factor MYC plays an important role in regulating the bioactivity of cancer stem cells. EpCAM is a common biomarker of cancer stem cells [110]. Upon cleavage of EpCAM, the intracellular domain functions as a part of a transcriptional complex inducing c-Myc and cyclin A and E [111]. The EpCAM antibody can be regarded as a kind of MYC inhibitor. For example, the use of EpCAM antibody catumaxomab for the treatment of non-small-cell lung cancer shows high efficacy in reducing the expression of MYC and killing cancer cells [112]. The combination of EpCAM antibodies and chimeric CAR-T technology has been tested in phase I trials for various types of cancer, such as NCT02725125, NCT02915445, and NCT02729493. The combination of MYC inhibitors and CAR-T is an effective tumor immunotherapy, but there are still some limitations and challenges. For example, CAR-T cell therapy remains limited in the treatment of solid tumors such as adenocarcinoma and sarcoma, and in clinical trials of solid tumors, CAR-T cell therapies have exhibited limited efficacy and severe toxicity [113, 114].

### Combination of MYC inhibitors and MYC agonizts

The combination of MYC inhibitors with certain agonizts has also shown increasingly significant clinical effects. Common MYC inhibitors can be divided into the following groups: agents that block MYC-MAX interaction (10054-F4, MYCi361, and MYCi975), those that either block the binding of MYC-MAX to DNA or those that prevent the expression of MYC (BET inhibitor (JQ1), CDK7 inhibitor (THZ1), cardiac glycosides (bufalin, ouabain), and cytoskeletal disruptors, among others) [77, 78, 88].

ONC201 acts as an allosteric agonist of the mitochondrial caseinolytic protease P, which can indirectly activate myc. Downstream of target involvement, ONC201 activates the ATF4/CHOP-mediated integrated stress response, which leads to TRAIL/death receptor 5 (DR5) activation and inhibition of oxidative phosphorylation via active MYC [115]. ONC201 has been demonstrated to prevent sphere formation and growth of colorectal cancer cells [116]. Integrins are a series of adhesion receptors. Inhibition of integrin CD11b can induce MYC expression, thereby promoting bone marrow cell polarization and tumor growth. Leukadherin1 (LA1) is used for the pharmacological activation of CD11b, which can synergistically inhibit the growth of CL66-LUc breast tumors and human MDA-MB-231 breast xenograft tumors [117].

SR1078 modulates the conformation of RORγ in a biochemical assay and activates RORα and RORγ driven transcription. SR1078 stimulates the expression of endogenous ROR target genes in HepG2 cells that express both RORα and RORγ [118]. Stimulation with the agonist SR1078 effectively blocks MYCN-mediated tumor growth and de novo lipogenesis and sensitizes NB tumors to conventional chemotherapy. In neuroblastoma patients, higher caspase-mediated apoptosis can be seen after SR1078 treatment, which allows a certain degree of recovery [119].

The combinations are not limited to the above agonizts and MYC inhibitors, but the combinations mentioned demonstrate the advantages of this therapy, which can more fully inhibit and regulate tumor growth through different pathways and channels.

## Conclusions and perspectives

Most human cancers have an abnormal expression of MYC, which can affect tumor immunity in the TIME, and thus, it is beneficial to research and develops drugs that act on MYC. In recent years, with increased in-depth research on MYC, scientists have successfully developed drug-based strategies to block tumor growth by inhibiting MYC, a key node in tumor immunity. This approach has many advantages for tumor treatment, such as inhibiting tumor proliferation and blocking the interference between tumor cells and immune cells in the TIME. All these effects suggest that MYC has the potential to become a therapeutic target in cancer.

To date, immunotherapeutic strategies targeting MYC mainly include inhibiting the translation of MYC, reducing the stability of MYC mRNA in tumors, and directly or indirectly inhibiting the activity of MYC. These methods aim to inhibit MYC directly using peptides, small molecules, and certain compounds or indirectly by blocking upstream or downstream signaling pathways such as the WNT/β-Catenin pathway.

Moreover, to study the treatment strategies for various cancers and to overcome tumor heterogeneity, researchers are now trying to establish combined immunotherapy approaches. The combined blockade of MYC and immune checkpoints has shown encouraging results in many early clinical trials. The combination of MYC and CAR-T therapy is gradually achieving a precise attack of tumor markers. Some agonizts combined with MYC inhibitors can more fully inhibit the proliferation of tumor cells through different pathways. Although scientists have achieved great results, the clinical results require further validation.

In conclusion, therapeutic strategies against MYC have been demonstrated by ample studies to be beneficial to the organic recovery of antitumor immunity, which provides a new avenue for cancer immunotherapy.

## Data Availability

All data generated or analyzed during this study are included in this published article.
